# Thoroughness of philosophy and return to religion in Tanabe Hajime

**DOI:** 10.12688/f1000research.130602.1

**Published:** 2024-01-12

**Authors:** Hiroto Doi

**Affiliations:** 1 Faculty of Humanities and Social Sciences, University of Tsukuba, Tsukuba, Ibaraki, 305-8571, Japan

**Keywords:** Tanabe Hajime, absolute religion, world religion(s), Platonism

## Abstract

Japanese philosopher Tanabe Hajime (1885–1962) not only discussed religion in ways that were rooted in the fundamental claims of his writings, such as Shinran in *Philosophy as Metanoetics* (1946), but he also made proposals on religion, as in *Demonstration of Christianity* (1948), where he advocated for the need for a “second religious reformation.” For Tanabe, philosophy and religion (along with science) are fused in a specific way, and it is possible to find a vision of religion to be aimed for that is worth considering as a theory of religion among Japanese philosophers. To examine the theory of religion in Tanabe, this study focuses on the work, *Demonstration of Christianity,* and other works from the perspective of religious studies. In *Demonstration of Christianity*, the concept of absolute religion toward the second religious reformation is envisioned based on Tanabe’s dialectics of the absolute mediation, and a discussion on his world religion can be found, although it is limited to Christianity and some Buddhist sects. Reflecting his absolute dialectic, Tanabe says that this world religion was established through the absolute mediation of Christianity with “ *Nembutsu-Zen*” (the unification of Pure Land Buddhism and Zen Buddhism). One cannot deny the impression of Christocentrism in these discussions, but they are in fact the result of Tanabe’s thoroughness in philosophy. What can be drawn from these discussions is that Tanabe's theory of religion is strongly connected to Platonism, which he considered the root of his dialectics. For example, by the third article in *Existence, Love, and Practice*(1947), “Self-Transcendence in Platonism and Faith in Gospel.” Tanabe’s argument reveals the characteristics of conceiving a new religion that combines Eastern and Western ideas, philosophy, and religion through a creative interpretation of Platonism, the source of Western thought where philosophy and religion are inseparably linked.

## Introduction

Nishida Kitarō (1870–1945), a well-known Japanese philosopher, and Tanabe Hajime (1885–1962), ^1^ who was influenced by Nishida but later criticized his philosophy, were known for their originality and deep thoughts on not only philosophy but also on religion. The fourth section of Nishida’s *An Inquiry into the Good* ( *Zen no Kenkyū,* 1911) is titled “Religion”, and it is often noted that Zen Buddhism has substantially influenced him. ^2^ His lecture notes also suggest that he was aware of contemporary theories on religious studies in the Western world. ^3^ Although Tanabe did not adhere to any particular faith, ^4^ religion was fundamental to his thinking. His interests shifted from Zen to Pure Land Buddhism, then Christianity, and again Zen Buddhism. Although this attitude is sometimes considered to indicate Tanabe’s lack of a core religion (like Zen in Nishida), it also signals the purity and thoroughness of his philosophical speculations. ^5^ The theme of religion is crucial to both Nishida and Tanabe.

A particular characteristic of Tanabe’s philosophy is the appearance of religion at major points in his treatises, as in the case of Shinran in *Philosophy as Metanoetics* ( *Zangedō to shiteno Tetsugaku,* 1946). ^6^ In his *Demonstration of Christianity* ( *Kirisutokyō no Benshō,* 1948), ^7^ he advocates for the “second religious reformation” (THZ 10, 11, 17, pp. 319–320). Tanabe’s proposals on religion are so aggressive to seem unusual for a philosopher. In his vision, philosophy and religion join. As he states, “Religion and philosophy have different starting points and paths, but in the end, they come to a point of unity from opposite directions” (THZ 11, p. 430). In Tanabe’s work, philosophy and religion (and science) exhibit a fusion—not simply an identification, but a mediation in his dialectic— ^8^ which reflects his ideal conception of religion. This view suggests that Tanabe’s thought is worth considering as one of the Japanese philosopher’s theories of religion.

The aim of this study is to examine Tanabe’s theory of religion, which is unique among Japanese philosophers, by first examining the absolute religion related to the second religious reformation as a keyword. However, the question arises as to how his unique absolute religion pursues a universal theory of religion. Therefore, we examine Tanabe’s world religion and investigate the elements that are underlying it. Through this process, this study helps clarify the uniqueness and significance of Tanabe’s theory of religion from the perspective of religious studies.

## Methods

To achieve the aforementioned research objectives, we examine various documents by the Japanese philosopher Tanabe, from the perspective of religious studies. Although Tanabe has numerous writings, our chosen scripts are limited to later works containing relatively further developed theory of religion. This study first examines *Demonstration of Christianity*, which calls for absolute religion and anticipates a second religious reformation, thus clearly showing Tanabe’s attitude towards the future of religion and its formation. However, as the title suggests, the book may seemingly have a close affinity to Christian theology. For a discussion not limited to Christianity, but open to other religions, Tanabe’s other works need consideration. We then discuss *Existence, Love, and Practice* ( *Jitsuzon to Ai to Jissen,* 1947). The focus of this study is on how Tanabe develops a theory of religion not limited to Christianity. For this purpose, the third article in the same book, “Self-Transcendence in Platonism and Faith in Gospel,” containing the corresponding argument, is selected for our study.

The basis of this study is the textual interpretations, but the author also focuses on letters and recollections that clues the understanding of how Tanabe constructed his theory of religion. The literature that is referred to, comprise of papers focusing on Tanabe’s later works. The perspective of this study’s analysis is Tanabe’s view of Platonism. One reason for adopting this, is the author’s previous research on Plato and Neoplatonism. In addition, Platonism crucially bridges philosophy and religion in Tanabe’s work. To examine his theory of religion without limiting it to a specific religion, it is presumably insufficient to compare Tanabe’s views on a specific religion with its doctrines. Focusing on Platonism and analyzing its relation to religion, is a necessary process to find a theory of religion in Tanabe’s works, that is not limited to a specific religion.

Based on the above discussion, we explore how Tanabe’s contemplation of religion is also a practice of forming religion, by referring to Platonism as a model for bridging philosophy and religion in Tanabe. ^9^

### Range of Tanabe’s discussion and religion in Japanese philosophers

Although religion has an important role in Tanabe’s thoughts, he states, “Since the principle of nothingness in the dialectic is the principle of philosophy, philosophy must come to it by negating itself, and must be restored to the philosophy of religion as an occasion of negation as religious faith” (THZ 9, p. 335). In other words, Tanabe’s thoughts focus on the philosophy of religion, not religion itself. ^10^ When addressing the religious aspect of Tanabe’s work, previous studies have mainly discussed it as a philosophy of religion from the perspective of a study of Japanese philosophy. ^11^ Few authors have examined it from the perspective of religious studies.

The word *syūkyō* (宗教) was first used in Japan in the early Meiji era as a translation of “religion.” In Japan, religion is the product of various discussions and attempts, mainly by intellectuals with religious backgrounds, such as Buddhism and Christianity, in turbulent circumstances. ^12^ However, previous studies have not fully clarified the relationship between these debates and the views expressed by Japanese philosophers. The study of this relationship falls between the analysis of Japanese philosophy, which attempts to understand the philosopher’s thoughts from within, and the study of religion, which addresses intellectuals with religious beliefs. ^13^

There are several possible reasons why the theory of religion in Japanese philosophers has not been well studied. First, although both Nishida and Tanabe established their scholarly careers at a time when the concept of religion was still being formed in Japan, their use of the word “religion” does not seem very strange from a modern perspective. It is conceivable that this makes it unnecessary for us to be very aware of how their concept of religion was defined. Another possible factor is that Nishida and Tanabe both mainly focused on so-called world religions, such as Buddhism and Christianity, and less on folk religions and practices. ^14^ Of course, one could criticize this point of view by saying that the two Japanese philosophers overlooked areas that do not belong to world religions or “higher” religions with established doctrines and scriptures. One could also argue that because this is a philosopher’s theory of religion, it is not necessary to discuss religions other than well-established world religions. With Tanabe in particular, compared to Nishida, there is little attempt to take a reflective consideration of religion itself. However, this does not mean that there is no need to examine the theory of religion in Tanabe, but rather that there are still areas that have not been explored by previous studies.

Concerning Tanabe, it seems significant today to consider the religion that he contemplated and attempted to form as part of his practice. This is because, while the religious intellectuals of his time were struggling to establish a new religion for a new era, Tanabe was seeking to do so from a different point of view. Tanabe’s discussion of religion is unlikely to be limited to the acceptance of the religious views of the time but is assumed to be unique. This is because Tanabe’s thoughts, expressed in the symbolic form of the vortex ( *kadō* 渦動), denies being static or fixed, and always places himself as a practitioner in dynamism. This study sheds light on aspects of Tanabe’s unique religion that have not yet been examined from the perspective of how religion is bridged by philosophy.

### Tanabe as a “practitioner”

To examine Tanabe’s concept of “religion,” we acknowledge how strongly religion is considered in his theory. If Tanabe considered religion weakly, it would have been difficult to discuss his theory of religion alongside the arguments made by the religious intellectuals of his time.

One of Tanabe’s major characteristics as a philosopher is his emphasis on practice ( *praxis*) rather than contemplation ( *theōria*). His practice does not refer to acts such as Zen meditation in Nishida. Still, it reflects his thorough contemplation of religion dialectically as a practitioner of philosophy ^15^ rather than a mere scholar. Therefore, even if it does not involve specific religious acts, such as rituals, Tanabe’s way of life and attitude may only be described as *praxis.* After retiring from Kyoto Imperial University, Tanabe moved to Kita-karuizawa, known for its cold climate in winter. Here, he devoted himself to philosophical research and writing. His disciples were concerned about his health and suggested that he move to a warmer climate, but he disagreed. ^16^ As an example of his philosophy in practice, Tanabe moved his life to a cold region as a punishment for his former actions. In addition, during Tanabe’s tenure at Kyoto Imperial University, he was criticized by Minoda Muneki and others, who were known as nationalists and attacked many scholars in the humanities. However, Tanabe published his rebuttal in their magazine ^17^ and engaged in a direct dialogue with Minoda. This episode shows Tanabe’s sincerity, which contrasts with Nishida’s advice ^18^ not to deal with Minoda. Tanabe put himself at risk by engaging with these aggressors. ^19^ Hence, although human factors are involved, his response to Minoda and others ^20^ demonstrates the practical side of Tanabe’s speculation.

Tanabe’s thoughts are also oriented toward transforming society. During the war, his insistence on “the logic of species” was fraught with danger, as it could be interpreted as a message that the nation should be respected. ^21^ Nevertheless, Tanabe’s pursuit of a thorough practice-oriented philosophy includes religion as an object. Hence, exploring his thoughts as a Japanese philosopher’s theory of religion is of great significance.

### Absolute religion in Tanabe

Tanabe’s vision is explicitly expressed in *Demonstration of Christianity.* As Itō points out, ^22^ this work shows Tanabe’s intention to form a new religion ^23^ oriented toward social practice by extending *Philosophy as Metanoetics*, which lacked socially oriented practice. Published in 1948, the book begins with a somewhat distinctive introduction in which Tanabe recollects his entry into high school (the old school system). ^24^ At that time, the First Higher School ^25^ students were confronted with the “inevitable problem” of Christianity, and Tanabe was no exception. This phenomenon was due to the influence of Christian intellectuals such as Uchimura Kanzō (1861–1930), Uemura Masahisa (1858–1925), and Ebina Danjō (1856–1937). Their influence was more significant than those of Buddhist intellectuals such as Kiyozawa Manshi (1863–1903) and Chikazumi Jōkan (1870–1941). Thus, Christianity “responded even better to the humanist demands of the youth” (THZ 10, p. 4). A science student at the time, Tanabe was not convinced by “mythical content that seems to be the product of fantasy, incompatible with scientific attitudes” (THZ 10, p. 6). Even after listening to lectures by Raphael von Koebel (1848–1923) and Hatano Seiichi (1877–1950) in university, he did not become a Christian. However, after his “Metanoetics” ( *zangedō*) lecture in 1944, ^26^ which culminated in *Philosophy as Metanoetics*, Tanabe “felt as if his eyes had been opened for the first time to the truth of the Christian gospel which I had not been able to see before” (THZ 10, p. 8). He decided to “confront Christianity which had been his longstanding issue” (THZ 10, p. 9) and wrote *Demonstration of Christianity.*

This work develops from the question “Jesus or Paul?” by William Wrede (1859–1906). The argument that Christianity, Marxism, and Japanese Buddhism, which are dissimilar, mediate and unite as a dialectic is, from today’s perspective, a reflection of the world at the time. Since this study focuses on the development of religion in Tanabe, we begin with “Appendix: Christianity, Marxism and Japanese Buddhism,” in which the theme appears several times. Although *Demonstration of Christianity* was published in 1948, this appendix was published the previous year, in 1947, and could be considered a stronger reflection of Tanabe’s awareness of the issues. In this appendix, Tanabe’s reference to the second religious reformation and absolute religion ^27^ stands out as a remarkable theory of religion.

If Protestantism ends its protest halfway, it will naturally lose its life. It must first die to itself for its resurrection. It must live in nothingness with a thoroughgoing self-negation. This is Christianity, but no longer Christianity. But it is not Buddhism, of course, and it can only be called absolute religion or absolute faith. I believe that it is in this position that the second religious reformation is required to be carried out today. (THZ 10, p. 319)

Although the second religious reformation that Tanabe discusses has Protestantism in mind, ^28^ it is also mediated by Buddhism through negative occasions, becoming an absolute religion that “is Christianity but is not Christianity.” As Liao points out, the “absolute” in this absolute religion is derived from Georg W. F. Hegel ^29^. Therefore, Tanabe’s absolute religion is based on Christianity, the religion of history, and Christianity’s position as an absolute religion that is a “religious faith through a concrete historical mediation” (THZ 10, p. 322).

… I am sure that everyone is aware that the concept of absolute religion mentioned above is derived, of course, from Hegel. My use of it is the same as his, and while it involves historical mediation to a great extent, it must also exhibit the character of nothingness more thoroughly than it did in his case. If you think about it for a moment, absolute religion might seem to refer to a religion such as Zen, which absolutely denies any limitation and is universally free and flexible. Such universality, however, remains a so-called abstract universality, not a concrete universality. (THZ 10, p. 320)

Buddhism is not given sufficient significance in Hegel’s theory of religion in the *Phänomenologie des Geistes.* However, in this quote, Tanabe emphasizes the negative occasion of nothingness that appears in Zen and other forms of Buddhism and tries to give it a role by concretely and universally establishing absolute religion. After pointing out that “it is also clear that, relying on today’s developments in religious studies, Buddhism represents the position of nothingness as opposed to the position of being in traditional Christianity, and is the essential occasion for the mediation of the truly dialectical absolute religion” (THZ 10, p. 323), Tanabe goes on to state:

It must be said that only by including this can absolute religion truly become absolute. For without the occasion of nothingness, the absoluteness of religion cannot be realized *against itself.* In this sense, Mahāyāna Buddhism is truly representative of the absoluteness of absolute religion. However, as we have often seen, the absolute must be mediated by the relative, or else it loses its absoluteness. To demonstrate its content as an absolute mediation in the modern age, Buddhism must not simply return to the past, but rather be rooted historically, mediated by the religious needs of the present. (THZ 10, p. 323)

Tanabe advocates a thoroughgoing absolute religion from the standpoint of a philosophy of religion based on Hegel’s theory of religion by adding the Buddhist concept of nothingness to the dialectic of that philosophy. Tanabe’s absolute religion, with Christianity in mind and Buddhism added to it, differs from Hegel’s view of the history of religions. However, this reference to absolute religion, characteristic of Tanabe’s theory of religion, does not clarify what kind of a universal religion he aims for. Hence, we address Tanabe’s reference to “world religion(s)” ^30^ in our discussion.

### World religion in Tanabe

Absolute religion is used in Tanabe as if it were a synonym for world religion. In his lecture, “The Limits of Culture,” for example, Tanabe states, “The question of death forms the central problem in all religions, especially in Buddhism and Christianity, which are called absolute or world religions” (THZ 8, p. 281). ^31^ World religions in Tanabe are not based on population or regional distribution but are limited to “Buddhism and Christianity, which today are recognized as two opposing world religions” (THZ 11, p. 447). Previous studies also identify Buddhism and Christianity as world religions in Tanabe. ^32^ Furthermore, Christianity is representative of world religions in his works. Paul superseded Judaism, a tribal religion (THZ 10, p. 66), and became the driving force in establishing Christianity as a world religion (THZ 10, pp. 174, 237). ^33^ An essential element in this world religion is that it “is not concerned with the comfort of self alone, but intends the comfort and salvation of all humankind” (THZ 10, p. 147), namely, it “advocates the salvation of humanity” (THZ 8, p. 432). Tanabe also states that “all world religions recognize love at the root of existence, and they generally preach love as the principle of spirituality as the highest being of human” (THZ 13, p. 189).

From these assertions, it would seem that Tanabe is simply defining world religions based on Christianity. Passages such as “there can be no religion apart from theology in world religions” (THZ 10, p. 255) seem to confirm this view. However, in his later work, *Introduction to Philosophy,* Tanabe not only states that theology that “organizes the content of the beliefs of some particular religion into a logical doctrine” is essential for evangelism but also that it is appropriate for “a theology that attempts to logically formulate the absolute universally” to be called a philosophy of religion if it moves beyond the specifics of particular religions (THZ 11, pp. 38–41). Hence, in Tanabe’s view, world religions are defined by the degree to which they reflect his dialectic. ^34^ From the standpoint that “it is essential to clarify the essential differences between Buddhism and Christianity and the point of unity between the two religions from the standpoint of religious philosophy” (THZ 10, p. 451), Tanabe argues the ideal religion as below. ^35^

Pure Land Buddhism, which is included in Nāgārjuna and other branches of Mahāyāna Buddhism, shows that Buddhism as a religion cannot lack the occasion of salvation by Other-power ( *tariki* 他力). The fact that it is thoroughly realized as the position of nothingness in Zen on the one hand, and the return to the love of grace by Other-power on the other, can be understood as the reason for the realization of the essence of the religion of nothingness as love and love as nothingness. In this sense, Zen and Nembutsu should be regarded as opposing occasions that establish the religiosity of Buddhism. If a concrete synthesis of the two, which may be called Nembutsu-Zen, ^36^ is realized, it can be understood as the most concrete and universal stage of Buddhism. However, it is a logical necessity that the creative development of such a synthesis requires a religious form corresponding to the historicity of the particular, mediating between the universal of nothingness and the individual of love. To think that this is offered in Christianity should be expected from what has already been said. In the mediation of these three, the unifying content of absolute mediation is embodied, and this alone is the task of religious innovation at present. (THZ 10, p. 34)

It is not difficult to imagine that the absolute mediation of Zen, Pure Land Buddhism, and Christianity would result in the aforementioned absolute faith or religion, but one cannot deny the impression that the philosopher Tanabe is creating a religion by cherry picking. Tanabe’s attitude toward including religion in the philosophical discussion became more pronounced after the *zangedō* period. In “The Logic of Place and the Religious Worldview ( *Bashoteki Ronri to Shūkyō-teki Sekai-kan*, 1945),” written shortly before his death, Nishida’s statement that “a philosopher should not fabricate religion based on his system” ^37^ may be considered a reflection of Tanabe’s attitude. ^38^ How can a philosopher form a religion, whether fabricated or not? As noted above, the main theme of *Demonstration of Christianity* is not the formation of religion by philosophers or the bridge between philosophy and religion. The book is a realization of Tanabe’s long-considered confrontation with Christianity in his unique demonstration. However, this awareness of the problem can be traced back to Tanabe’s earlier writings, which are also discussed at the beginning of *Demonstration of Christianity.*

I have already mentioned that I have come to feel a kinship with Christianity since I was converted to “philosophy as metanoetics.” Moreover, when I reflected on the dialectic of Platonism that I had adopted in the past, it became clear to me that I could no longer stop there. I then became aware that it was inevitable that I should go on to the position of paradoxical dialectics and come to faith in the gospel. In other words, the dialectic of good must now be turned into the dialectic of love. (THZ 10, pp. 11–12)

Here, “the dialectic of Platonism that I had adopted in the past” is a clear reference to the third article in *Existence, Love, and Practice*, “Self-Transcendence in Platonism and Faith in Gospel.” In addition, with the “dialectic of good,” he probably has the “idea of good” in Plato’s *Republic* in mind, extending it to the Christian dialectic of love and Buddhism. As noted earlier, world religions are based on love (THZ 13, p. 189) as a requirement for the salvation of humanity (THZ 8, p. 432). Therefore, *Existence, Love, and Practice* seems to suggest how philosophy is connected to the formation of world religion and its universality from Tanabe’s standpoint. In the next section, we will examine how his conception of world religion is bridged through philosophy.

### Platonism as a framework for world religion in Tanabe

The third article in *Existence, Love, and Practice*, “Self-Transcendence in Platonism and Faith in Gospel,” is vital for understanding how philosophy is connected to religion in Tanabe. The article opens with a chapter titled “Socrates’ Tragic Death Hymn and Philosophy,” which discusses Socrates’ “practice of death” as depicted in Plato’s *Phaedo*:

In philosophy, we attain the wisdom of liberation through the path of nothingness. Its starting point is in accord with the criticism of science, and its point of return is in accord with religion as a standpoint for liberation and salvation…. To die, that Plato demanded of the true philosopher, must be to stand in such a standpoint of nothingness. (THZ 9, pp. 405–406) ^39^

The unity of the point of return of philosophy and religion is presented by Socrates. Then, *daemōn*’s voice to Socrates is explained as the intervention of the Other-power, which urges repentance as absolute conversion. Tanabe asserts that “philosophy must be the very practice of absolute nothingness” (THZ 9, p. 430). In *Demonstration of Christianity*, the occasion of absolute nothingness through the death and resurrection of Jesus is pointed out as the reason why Judaism, which remained within the bounds of tribalism, was transcended and became a world religion called Christianity. Zen is also a religion of nothingness for Tanabe, and Pure Land Buddhism is a religion of Other-power salvation. Thus, “Self-Transcendence in Platonism and Faith in Gospel” refers to Socrates in the same way as the world religions in *Demonstration of Christianity.* Moreover, since Socrates is also regarded as an ordinary man ( *bonbu* 凡夫) who obtains salvation through repentance ( *zange*懺悔) in Other-power religions (THZ 9, p. 29), his practice of death comes even closer to salvation through Other-power religions as the only way possible, even for ordinary people.

“Self-Transcendence in Platonism and Faith in Gospel,” which begins with the death of Socrates, discusses such themes as the development of Plato’s Idealism, the significance of matter as an occasion of self-negation in Plato’s philosophy, its similarity to Buddhism, and the fact that the existential philosophy of the time, including Karl Jaspers, became an overreach of Plato’s dialectics as well as Neoplatonism. Toward the end of the work in chapter six, “The Mediation of Zen of Learning Death in Nothingness as Love,” Tanabe refers to Suzuki Shōsan in the early Edo period to make practicing death possible even for ordinary people. This argument may seem rather far-fetched, but Tanabe sees in it a connection between philosophy and religion that transcends both East and West. Suzuki Shōsan was a Zen monk of samurai origin and was regarded as an advocate of economic ethics due to his insistence that daily life was a Buddhist practice. Tanabe contrasts Suzuki’s experience of constantly facing death with that of Socrates and interprets it as follows:

I find it very significant that on the method of learning death, we can recognize the process by which philosophers practice death, something that Socrates seems to have accomplished with almost superhuman innate gifts and training. … Therefore, if we learn death by dying in death, as Shōsan dōjin has shown, then true death, no longer an object of conception, becomes the inner occasion that makes us subject. This learning from death is the only way to practice death that is open to us ordinary people. (THZ 9, pp. 473–474)

Furthermore, Tanabe, referring to Suzuki Daisetsu, interprets the emphasis on knowing shame as a characteristic of Zen of learning death as “an emphasis on repentance not normally found in Zen” (THZ 9, p. 474). He praises Shōsan Zen as “deeply committed to the absolute state of religion, without any doubt” (THZ 9, p. 475). However, Zen was a standpoint pursuing absolute nothingness through self-power practice and treating others with love was thin and abstract (THZ 9, pp. 477–478). Here Tanabe states that by applying his dialectic principle to Zen, the Zen of self-power and the Nembutsu of Other-power go together to arrive at the original state of being.

In this way, the belief in self-power, a characteristic of Zen, becomes *against itself* through transcendence as immanence and is developed into self-power as Other-power through the mediation of the method learned from death. Thus, combined with the social nature of love mentioned above, Zen’s demand for self-centered liberation, as rooted in the mercy of Buddha and the fundamental vow of Tathāgata, is transformed into gratitude for the other and proceeds to the phase of returning to the other. (THZ 9, pp. 478–479)

In Tanabe, because repentance by Other-power is the essence of Shinran, Suzuki Shōsan’s Zen practiced by ordinary people who repent may be connected to Nembutsu (THZ 9, p. 476). Hence, with Socrates as a starting point, Zen and Nembutsu are strongly linked, and religious salvation is not limited to self-salvation but is also linked to social practice. This also corresponds to the fact that Plato’s dialectic, which began with Socrates, is oriented toward self-transcendence and necessarily leads to social practice, hence the claim that Platonism is completed by the Christian gospel.

In seeking a satisfactory and possible way to meet the inevitable demand of the Platonic dialectic to transcend the self through dialectical methods, I have taken up and interpreted, and critiqued ideas such as existential philosophy and Zen of learning death, which have much in common with Plato’s philosophy. I realized, however, that these are still one-dimensional abstractions and that we must demand the opposite positions of social liberation and Other-power return as an occasion, and that they must also be mutually combined through them. Thus, as a concrete religion that mediates and integrates these two or three opposing occasions, I have no choice but to take up faith in the gospel. (THZ 9, p. 481)

This statement also seems to be an assertion that world religions, including philosophies, are unified under Christianity as the standard. However, as discussed in *Demonstration of Christianity*, the element of dialectic mediated by the negative occasions allows Christianity to break away from the tribal religion of Judaism to become a world religion. In the same book, Plato is indicated as the founder of that dialectic (THZ 10, p. 28). Plato’s later philosophy establishes the dialectic common to Christianity that practically mediates individual existence toward the social organization (THZ 10, p. 29). Although one cannot deny the impression of Christocentrism, Platonism can be seen as the framework for the world religion that Tanabe calls for in the second religious reformation, in which Christianity and Buddhism are linked. In other words, Platonism, as the root of dialectics, is the mediator between philosophy and religion and the condition for forming what Tanabe calls a world religion.

This insistence on moving from Platonism as a dialectic philosophy to a world religion that must include a dialectical occasion is also found in *Dialectic of the Logic of Species*, published in 1947. However, there are few references to religions other than Christianity. In this work, published slightly earlier than *Existence, Love, and Practice*, Plotinus is frequently mentioned critically for his emphasis on contemplation. In contrast, Plato is mentioned positively for his emphasis on practice. However, Tanabe also noted that the person who best understood and succeeded Plotinus was Meister Eckhart, known as the Christian mystic of medieval Germany, who overcame Plotinus by emphasizing practice (THZ 7, p. 310). ^40^ As in “Self-Transcendence in Platonism and Faith in Gospel” from *Existence, Love, and Practice*, when Platonism is correctly and thoroughly implemented in Tanabe’s dialectic, we observe a structure that leads to a world religion with Christianity as its main axis.

## Conclusion

In this discussion, we have seen that in the Japanese philosopher Tanabe’s theory of absolute dialectic, the way of absolute religion is envisioned for the second religious reformation, and that there is a discussion of world religions, though limited to Christianity and some sects of Buddhism. The religion of the new era as envisioned by Tanabe, is a universal one in which philosophy and religion are bridged through dialectics and where Platonism is presented as a model for realizing this. Tanabe’s discussion reveals the following aspects that have not received much attention so far. The Japanese philosopher who confronted Western philosophy with intensive contemplation made a “creative” ^41^ interpretation of Platonism as an idea that inseparably links philosophy and religion and envisions a religion for a new era connecting East and West, philosophy, and religion.

Tanabe’s conception of religion probably does not go beyond the scope of an intellectual’s religion. The scope of his discussion of world religions is narrow, and his attention to folk customs and practices is limited. This would indicate that even if Tanabe’s thoroughness in philosophy had led him to religion, he would not have escaped the limitations of a “religion” that is mainly based on doctrinal and speculative aspects. However, this does not mean that Tanabe’s inquiry was not directed toward practices such as folk customs that today’s religious studies would naturally include in the realm of religion, but rather that Tanabe was directed toward practices as a philosopher. Although it may appear to be an empty theory, for Tanabe, it was an existential practice and not an empty argument as shown in *Demonstration of Christianity.* ^42^

The confrontation with Minoda also evidences the intensity of Tanabe’s determination to pursue his practice of philosophy. The fact that the thoroughness of his philosophy, on which he stakes his existence, is somewhat aligned with religion, may be considered a practice of retracing the path of Socrates, the founder of the dialectic, as Tanabe calls him. Platonism, which originates in the practice of death by Socrates, is, according to Tanabe, the driving force behind the development of Christianity into a world religion of humanity and a borderline position that may diverge into Neoplatonism as an overreach of Plato, which Tanabe criticizes. ^43^ Thus, Platonism in Tanabe’s philosophy, as a prototype of dialectics, is the basis of his philosophy. It also plays an interesting role as a mediation between his philosophy and world religions that aim at universal salvation. ^44^

Hatano Seiichi, whose lectures Tanabe also attended, wrote of Neoplatonism, including Plotinus, that “this school of philosophy sought to satisfy the truth-seeking mind on the one hand and the religious mind on the other. In a word, it is the religion of scholars,” in his *The History of Western Philosophy* ( *Seiyō Tetsugakushi-yō,* 1948). ^45^ Plotinus was a philosopher whom Tanabe criticized for his bias toward contemplation. Tanabe contended that Christianity had overcome it. Aside from “unification” ( *henōsis*), Tanabe and Plotinus may have something in common in that they have practiced philosophy with a religious mind in seeking salvation and that, in the end, the thoroughness of their philosophical inquiry has led them to religiosity. What does the thoroughness of the philosopher’s inquiry and its practice at a turning point bring to religion? As the above discussion shows, this question is of particular interest to Japanese philosophers. However, to examine this theme in Japan, the trend concerning (self-) cultivation ( *shūyō* 修養) should be considered, which was common among both scholars and religious intellectuals from the middle of the Meiji era. This point could not be dealt with adequately in this study and should be explored further in the future.

## Data Availability

No data are associated with this article.

## Footnotes

^1^
Tanabe’s work referred to in this study is based on *Tanabe Hajime Zenshū (Collected Works of Tanabe Hajime)*, Tokyo: Chikuma Shobō, 1976 (3rd. pr.), abbreviated as THZ. When citing or referring to the work, the reference is indicated by the THZ volume number and page number. The quotations in parentheses ( ) are the original text, and brackets [ ] indicate the author’s additions.

^2^
It has also been pointed out that Nishida’s strong influence from Zen has not always been fully demonstrated (Sakamoto Shin-ichi, “Nishida Tetsugaku no Futatsu no Keishō: Kyōto Gakuha to Chisan Gakuha (Two Inheritances of Nishida Philosophy: Kyoto School and Chisan School),” *Hikaku Shisō Kenkyū (Studies in Comparative Philosophy)*, No. 48, 2022, pp. 115–116).

^3^
The new edition of the *Collected Works of Nishida Kitarō*, Vol. 14, includes a record of Nishida’s lectures on religious studies. The section “The Rise of Religious Studies and Its Development” lists the scholars of religious studies since Max Müller. Takeda Atsushi, Klaus Riesenhuber, Kosaka Kunitsugu, Fujita Masakatsu (eds.), *Nishida Kitarō Zenshū (Collected Works of Nishida Kitarō)*, Tokyo: Iwanami Shoten, 2004, Vol. 14, pp. 37–42.

^4^
In *Demonstration of Christianity*, for example, Tanabe recounts his relationship with religion, including Christianity, and states that he never held any religious beliefs to maintain his position as a philosopher (THZ 10, pp. 3–9). In his *Introduction to Philosophy* *(* *Tetsugaku Nyūmon* *)*, published in 1951 and based on lectures given from 1948 to 1951, Tanabe stated that although he had no specific religion or belief, “I cannot suppress the religious urge to be aware of my relationship with something absolute” (THZ 11, p. 444). This attitude should not be taken as a suspension of judgment concerning value or a neutral attitude toward religion but rather as an indication of the thoroughness of his philosophy.

^5^
Himi Kiyoshi, *Tanabe Tetsugaku Kenkyū: Shūkyō Tetsugaku no Kanten kara (Studies in the Philosophy of Tanabe Hajime: From the Perspective of the Philosophy of Religion)*, Tokyo: Hokuju Shuppan, 1990, p. 20.

^6^
The year of publication may mislead readers into thinking that this work was written in response to Japan’s defeat in World War II. However, it is based on a lecture titled “Metanoetics” ( *zangedō*) given in 1944 after the setback of the logic of species ( *shu no ronri*), and it is mentioned in a commentary by Ōshima Yasumasa that the book was still in the process when Japan lost the war (THZ 7, pp. 382–384).

^7^
Tanabe distinguishes between the conscious “ *benshō* (辯証)” and the objectified “ *benshō* (辨証),” merely seen from the outside, and adopts “ *benshō* (辯証)” for the book’s title (THZ 10, pp. 38–40).

^8^
It should be noted that Tanabe’s dialectic is his theory, which he calls “absolute dialectic.” Therefore, when Tanabe uses the term “dialectic” to refer to Plato in particular, he includes many of his interpretations that depart from Plato’s text.

^9^
It is important to consider the division of Tanabe’s thoughts into periods, which differs among scholars. In this study, the main subject under consideration is Tanabe’s writings after his lecture “ *zangedō*” in 1944, which belongs to the period after which Tanabe realized the inadequacy of the logic of species. This later period of Tanabe is strongly characterized as a philosophy of religion. In line with Himi’s interpretation, this study takes the position that Tanabe maintains a consistent problematic awareness of religion, even though the subject matter he addresses changes over time (Himi, *op. cit.*, pp. 19–20).

^10^
It should be noted that Tanabe’s philosophy of religion has a different connotation compared to the philosophy of religion in general terms, such as Kant’s philosophy of religion, for example. Tanabe also refers to the philosophy of religion in philosophy in general, for example, in “Supplement III: Philosophy of Religion and ethics” in his *Introduction to Philosophy.* He states that the philosophy of religion is a touchstone for each philosophy to question its true value (THZ 11, pp. 429–432).

^11^
As works that thematically discuss Tanabe’s philosophy of religion, we can mention Himi, *op. cit.*, and Liao Chin-ping, *Shūkyō Tetsugaku no Kyūsai-ron: Kōki Tanabe Tetsugaku no Kenkyū (The Soteriology of Religious philosophy: A study of Tanabe Hajime’s Late Philosophy)*, Taipei: National Taiwan University Press Center, 2018.

^12^
For a study of these religious figures and their theories, see Hoshino Seiji, *Kindai Nihon no Shūkyō Gainen: Shūkyōsha no Kotoba to Kindai (The Concept of Religion in Modern Japan: Religious Figures and Modernity)*, Tokyo: Yūshisha, 2012.

^13^
The claims made by modern Japanese religious figures in Hoshino *ibid.* diverge from Tanabe’s theory of religion. This difference may be explained by a difference in positions: while some of the religious figures mentioned in the book approach philosophy from the perspective of religion, Tanabe identifies himself as non-religious and approaches religion from the perspective of philosophy.

^14^
In *Demonstration of Christianity*, Tanabe states that religion apart from theology cannot be a world religion and excludes indigenous beliefs from his consideration (THZ 10, p. 255). Citing Edward B. Tyler and Cornelis P. Thiele, Nishida also mentions primitive religions, and states that the essential classification of religions is not monotheism/polytheism or natural religion/revealed religion, but “according to the content of vital demand” ( *Collected Works of Nishida Kitarō*, Vol. 14, 2004, pp. 80–86).

^15^
Nakano Hajimu (ed.), *Kindai Nihon Shisō Taikei 23 Tanabe Hajime Shū (Modern Japanese Thought Series 23: Tanabe Hajime Collection)*, Tokyo: Chikuma Shobō, 1975, p. 449.

^16^
Tanabe’s consistent refusal to move to a warmer climate when advised by Karaki Junzō and others close to him are documented in letters to Karaki dated July and October 1947 ( *Tanabe Hajime Karaki Junzō Ōfuku Shokan (Tanabe Hajime and Karaki Junzō Reciprocal Letters)*, Tokyo: Chikuma Shobō, 2004, pp. 62–63, 72). For background on Tanabe’s stubborn stance, see the discussion by Itō Susumu ( *Ai to Shi no Tetsugaku: Tanabe Hajime (The Philosophy of Love and Death: Tanabe Hajime)*, Tokyo: Hokuju Shuppan, 2005, pp. 139-179).

^17^
Tanabe Hajime, “ *Minoda-shi oyobi Matsuda-shi no Hihan ni Kotau* (Response to Mr. Minoda’s and Mr. Matsuda’s Criticisms),” THZ 8, pp. 13–31.

^18^
Letters to Mutai Risaku and others ( *Collected Works of Nishida Kitarō*, Vol. 22, 2007, pp. 150, 170).

^19^
For the discussions between Minoda and Tanabe, see Uemura Kazuhide, *“Nihon” heno Toi wo meguru Tōsō: Kyōto Gakuha to Genri Nihon Sha (Conflict over the Question of Japan: Kyōto School and Genri Nihon Sha)*, Kashiwa Shobō: Tokyō, 2007, pp. 142-144.

^20^
As to why Tanabe responded to the debate, Ōshima Yasumasa speculates that one of the attackers, Matsuda Fukumatsu, may have once been a student of Tanabe (THZ 8, p. 466).

^21^
See Himi, *op. cit.*, pp. 111–117.

^22^
Itō, *op. cit.*, pp. 122–123.

^23^
Mutō Kazuo explains this as follows: “Tanabe did not advocate a new religion himself, so to speak, but rather sought a deep self-reflection of Christianity and Buddhism from the standpoint of philosophical demonstration and hoped for creative development originating from within them” (THZ 10, p. 333). Depending on one’s view of the distinction between philosophy and religion, it is difficult to say whether *Demonstration of Christianity* was an attempt to establish a new religion or a proposal for religion from the standpoint of philosophy. However, as the practice of philosophy that Tanabe aimed at was in line with religion (THZ 11, p. 430), we argue that it was a practice aimed at some form of religion from the standpoint of philosophy.

^24^
When Tanabe was a high school student, discourses on (self-) cultivation ( *shūyō* 修養) were being developed by Christian and Buddhist intellectuals. Some studies note that during this period, there was a movement among scholars of religion, such as Anesaki Masaharu, to search for a new form of religion from the perspective of cultivation (Kurita Hidehiko, “The Notion of *Shūyō* and Conceptualizing the Future of Religion at the Turn of the Twentieth Century,” *Religious Studies in Japan*, 2018, Vol. 4, pp. 74–75). However, the word *shūyō* rarely appears in Tanabe. The few examples are his letter to Karaki in December 1947 (Tanabe and Karaki, *op. cit.* pp. 76-77).

^25^
The school was known as the predecessor of the Collage of Arts and Sciences at the University of Tokyo and others, with its dormitories being famous for their self-governing system. This spirit had already faded by the time Tanabe entered the school (THZ 10, p. 3).

^26^
Liao interprets this as a religious experience that occurred to Tanabe during this period. The author agrees with Tanabe’s description from the perspective of religious studies (Liao, *op. cit.*, p. 44).

^27^
For a discussion of how absolute religion in Tanabe is constructed through his absolute dialectic, see Liao, *op. cit.* pp. 167-175. This study focuses on the aspect of absolute religion as a theory of religion rather than its dialectical construction, then then discusses how Platonism bridges between philosophy and religion in Tanabe.

^28^
Protestantism “dynamically testifies to eternity insofar as it constantly renews itself in creative repetition and innovative circulation” (THZ 10, p. 322), but even in Hegel’s time, such a spirit was lost. Hence, the second religious reformation must restore its originality, as Tanabe suggests.

^29^
Liao, *op. cit.*, p. 66, 72, 110.

^30^
A well-known study of world religions is the one by Masuzawa Tomoko ( *The Invention of World Religions: Or, How European Universalism Was Preserved in the Language of Pluralism*, Chicago and Lindon: The University of Chicago Press, 2005). Tanabe’s world religions are quite different from those discussed in the book, especially the issue of the plurality of languages, which was becoming well known at the time but seemingly not reflected in his books.

^31^
This lecture was given to the national elite, First High School students, the year before Japan’s defeat in the war. It should be noted, therefore, that the “question of death” here was also given in the context of total war, a situation of urgency quite different from those in which philosophy and religion are discussed today.

^32^
See Himi, *op. cit.*, pp. 113, 116. Regarding the narrow range of religions that Tanabe took up in his discussion of world religions (although Tanabe states in THZ 11, p. 448 that Islam, like Judaism, is not a world religion because it is unique to a particular people), Himi said that it was unavoidable given the state of research in Japan at the time, but that Tanabe’s failure to mention Shintoism, perhaps due to insufficient awareness of the issue of the Emperor System, greatly reduced his persuasiveness (Himi, *op. cit.*, pp. 182-183). The author speculates, in contrast, that Tanabe may not have placed Shintō in the category of religions that he can discuss, either concerning the so-called state Shintō or to Shintō as a custom. This may be attributed to the fact that Shintō was not considered a religion by scholars of the time, as discussed in a 1949 dialogue between Ienaga Saburō and Yanagita Kunio: (Ienaga) “Especially about Shintō, written thought is no good at all.” (Yanagita) “I am painfully aware of this” (Ienaga Saburō, *Tanabe Hajime no Shisōshi teki Kenkyū” Sensō to Tetsugakusha* ( *An Intellectual History of Tanabe Hajime: War and Philosopher)*, Tokyo: Hōsei Daigaku Shuppan Kyoku, 1974, p. 423). In any case, it is possible that Tanabe did not intentionally include Shintō in his discussion.

^33^
Tanabe’s interpretation of this establishment is also based on his dialectic (THZ 10, p. 90).

^34^
The death and resurrection of Jesus is a strong manifestation of this element. In *Demonstration of Christianity*, Tanabe states that “it would be impossible to find an equivalent occasion in Buddhism” (THZ 10, p. 35) and mentions other fundamental differences between Christianity and Buddhism (THZ 10, p. 48). However, despite the differences, the fact that a sect that lacks a dialectic cannot become a world religion and remains Japanese Buddhism (THZ 10, p. 309) is connected to the assertion that Buddhism is a world religion by his dialectic.

^35^
In Tanabe, Pure Land Buddhism is the closest to Christianity as a religion of Other-power salvation (THZ 10, p. 33), and Zen Buddhism is considered to have the occasion of nothingness corresponding to the death of Jesus.

^36^
For Nembutsu-Zen in Tanabe’s context and its dialectic construction, see Liao, *op.cit.*, pp. 84-91.

^37^

*Collected Works of Nishida Kitarō*, Vol. 10, 2004, p. 295.

^38^
Nakano, *op. cit.*, p. 457. In response to these points made by Nakano, Himi observes that since Tanabe is not named, “it would be sufficient to accept it honestly as a very general phrase of admonition.” Himi, *op. cit.*, p. 28. However, as Nakano explains, the following words of Tanabe in “I Ask for Professor Nishida’s Instruction” in which he turns to a criticism of Nishida, can be interpreted as a rebuke directed at Tanabe in his later years: “I cannot help but be suspicious of the religiousization of philosophy” (THZ 4, p. 328) and “I think that the religiousization of philosophy cannot avoid the tendency to make philosophy an imperfect imitation of religion, instead of recognizing its authority. By its very nature, philosophy aims at a standpoint free from all standpoints. Religion is also a standpoint, and therefore should be transcended” (THZ 4, p. 313).

^39^
Tanabe states that “Socrates was a hero and a superhuman, not a philosopher like Plato himself” and that “in philosophy, which is thoroughly concerned with the dialectic of consciousness of thought, he only taught its starting point and the direction it should go, but it cannot be said that he fully developed it” (THZ 9, p. 413), and that the dialectic was perfected by Plato who succeeded Socrates. He then positions the Neoplatonism of Plotinus and others as an “overreach” of Plato by transferring the negative occasion of nothingness to the position of life (THZ 9, pp. 92, 398–399, 432). This indicates that Tanabe regarded Socrates, Plato, and Plotinus as a continuous Platonism. Tanabe was referring to research trends in ancient Greek philosophy and was critical of the theory of the differences between the philosophies of Socrates and Plato (Burnet=Taylor theory) that was being proposed at the time (THZ 9, 411, pp. 414–415).

^40^
A similar statement is found in *Philosophy as Metanoetics* (THZ 9, p. 258).

^41^
The bracketed “creative” is because Tanabe’s argument is formed by including a misreading of Plato. An example is his interpretation of the “practice of death (Plato’s *Phaedo*)” in “Self-Transcendence in Platonism and Faith in Gospel” (THZ 9, p. 406).

^42^
“My demonstrations are inevitably criticized as philosophical fantasies by genuine Christians just as Buddhists call it an unserious argument, I must be willing to accept criticism from Christians that it is fanciful. I can only endure in despair but not despair, relying on the words, “he that shall endure unto the end, the same shall be saved” (Mark 13: 13). In that case, Paul’s conversion will be a great help to me. No, not only that. I believe that the truth of the dialectic is already being proven in the execution of this task, as it is an unavoidable philosophical demonstration for me, even if it is criticized by others” (THZ 10, pp. 92–93).

^43^
On the positive and negative influences from Plato and other Greek thought on Christianity as a world religion, see THZ 10, pp. 78–79.

^44^
After *Demonstration of Christianity*, in which Tanabe praised Christianity, especially Protestantism, he took a new turn by approaching Buddhism and Zen in particular. The awareness of the Master as a relative person that was developed in *Demonstration of Christianity* (THZ 10, pp. 82, 89) led to a shift from Christianity, which was limited to salvation by the Savior and Master, to *Bosatsudō*, in which the many relativities of ordinary people worked together to save one another. What made this development possible was probably Tanabe’s theory of world religions, which, from *Philosophy as Metanoetics* to *Demonstration of Christianity*, understood Zen as a religion of Other-power and connected it to Christianity. However, a discussion of this theme is beyond the scope of this study and should be explored in future research.

^45^
Hatano Seiichi, *Hatano Seiichi Zenshū (Collected Works of Hatano Seiichi)*, Tokyo: Iwanami Shoten, 1968, p. 150. Circle points were added on “the religion of scholars” in the original text.

