# Supplementary material for: Thoroughness of philosophy and return to religion in Tanabe Hajime
Source: F1000Res. 2025 Mar 10;13:59. Originally published 2024 Jan 12. [Version 2] doi: 10.12688/f1000research.130602.2 (PMC12586978; doi:10.12688/f1000research.130602.2)
Supplement: Peer review history

# Reviewer report: Reviewer response for version 2

**DOI:** 10.5256/f1000research.178568.r370321

**Published:** 2025-11-04

**Authors:** Seiji Hoshino

**Affiliations:** 1 Kokugakuin University, Tokyo, Japan

In my initial review, I mainly suggested three issues for further explanation and clarification.

 1) Context

 Regarding the context of this paper, the author revised the introduction, sufficiently clarifying its scope. Also, the author added Note 51, which presented the connection to the idea of shūyō as an issue (along with other issues) to be pursued in the future.

 2) Tanabe's scope

 I commented on Tanabe's scope for interacting with society, or on how to embody his "absolute religion" in this world. The author edited Section 4 and added Note 32 to elaborate on those issues, successfully explaining Tanabe's attitude towards society. The author also noted that Tanabe remained ascetic as a philosopher, which, to me, was a reasonable explanation. 

 3) Tanabe's practice

 I also commented on Tanabe's practice, which was related to the second issue mentioned above. By editing Section 4, the author added an explanation of what Tanabe perceived as practice. It was not a simple argument, but needed addition, for Tanabe emphasized the importance of practice in his writing. By the edited version, these issues were addressed more clearly.

 Regarding the other minor issues I had noted, the author earnestly addressed them and replied as much as possible, noting that some would be pursued further in the future. I am particularly curious about Tanabe's passive (or negative) attitude towards "religious studies" of his time. However, as the author noted, this will be a question for the future.

 Overall, the author successfully edited his manuscript to make it more consistent and clearer. Thus, I approved this edition for indexing.

Is the work clearly and accurately presented and does it cite the current literature?

Yes

If applicable, is the statistical analysis and its interpretation appropriate?

Not applicable

Are all the source data underlying the results available to ensure full reproducibility?

No source data required

Is the study design appropriate and is the work technically sound?

Partly

Are the conclusions drawn adequately supported by the results?

Yes

Are sufficient details of methods and analysis provided to allow replication by others?

Yes

Reviewer Expertise:

History of religions in modern Japan

I confirm that I have read this submission and believe that I have an appropriate level of expertise to confirm that it is of an acceptable scientific standard.

# Reviewer report: Reviewer response for version 2

**DOI:** 10.5256/f1000research.178568.r370320

**Published:** 2025-03-28

**Authors:** Ellie Palmer

**Affiliations:** 1 The University of Edinburgh, Edinburgh, Scotland, UK

I’m happy to approve this article.

Is the work clearly and accurately presented and does it cite the current literature?

Yes

If applicable, is the statistical analysis and its interpretation appropriate?

Not applicable

Are all the source data underlying the results available to ensure full reproducibility?

No source data required

Is the study design appropriate and is the work technically sound?

Partly

Are the conclusions drawn adequately supported by the results?

Yes

Are sufficient details of methods and analysis provided to allow replication by others?

Yes

Reviewer Expertise:

NA

I confirm that I have read this submission and believe that I have an appropriate level of expertise to confirm that it is of an acceptable scientific standard.

# Reviewer report: Reviewer response for version 1

**DOI:** 10.5256/f1000research.143372.r294163

**Published:** 2024-06-27

**Authors:** Ellie Palmer

**Affiliations:** 1 The University of Edinburgh, Edinburgh, Scotland, UK

**Paper Summary**

 This paper focuses on the apparently inextricable relationship between philosophy and religion in the writing of Tanabe Hajime, centred upon his later works, in particular “Demonstration of Christianity” in which Tanabe’s notion of “absolute religion” is expounded. The author of this paper takes this concept as synonymous to “world religion”, whilst acknowledging the restriction of Tanabe’s discussion to Christianity and Buddhism. The author’s focus lies in interpreting this combination of Buddhism and Christianity with Platonism as the “bridge” between Tanabe’s theories of philosophy and religion. The author claims this “bridging” finds its basis in Tanabe’s indication that Plato is the founder of his dialectic. It is this dialectic which forms the fundamental mediation between philosophy and religion that defines Tanabe’s theory of the philosophy of religion. The author defines world religion, in an interpretation of Tanabe, based not only on Christianity but by the degree to which they reflect Tanabe’s Platonic dialectic, concluding that it is Platonism, despite the apparent Christocentrism in Tanabe’s work, that can ultimately therefore be considered the framework for Tanabe’s concept of world religion and, further, is itself a world religion.

**Contribution to field**

 This paper makes a unique and valuable contribution to the field by choosing to examine not merely Tanabe’s theory of religion in isolation but specifically acknowledging it within the context of Tanabe’s reading of Platonism and the inseparable connection to his wider philosophy. The author also engages in discussion of more specific concepts in Tanabe’s philosophy of religion that are, in fact, key to its understanding yet often overlooked, such as the notion of an “absolute” or “universal” religion and the “second religious reformation” discussed in Demonstration of Christianity. In general, Japanese philosophy, including Tanabe’s theory of religion is frequently examined in this comparative context between “east” and “west”, and other times, as the author points out, exclusively from the perspective of Japanese philosophy. Interestingly, however, this paper situates Tanabe’s theory of religion as grounded in his interpretation of Platonic theory. This arguably makes for a stronger foundation to both portray and discuss Tanabe’s theory, rather than evaluating Tanabe’s theory in reference to a particular religion or tradition, or being tempted by applying this to a comparison of eastern and western religion, which would fail to preserve Tanabe’s fundamental aim of establishing a universal theory of religion. Discussion and development of Tanabe’s theory of religion is a particularly welcome topic today, where the world is increasingly secular, yet many still yearn for faith and the societal and meaning-based role that religion plays in life in the way Tanabe describes through his work on the philosophy of religion.

**Recommendations**

- Content

I’m not entirely convinced whether the section on Tanabe as a “practitioner” adds much to the overall paper. The idea that Tanabe’s philosophy of religion required a certain “practice” is relevant but why this is was not entirely clear within this particular section. This section could be expanded in terms of what this practice entails and how the theories the author discusses should be employed in a wider societal, practical and ethical context, and/or condensed in terms of the background content.

- Context and scope

The author mentions throughout this paper the fact that Tanabe’s theory of religion was not only extremely “unique” or “unusual”, but even radical and “aggressive”. It would be beneficial to draw on precisely why this was, through situating Tanabe’s work within some further discussion and analysis of the previous attitudes and views on the link between philosophy and religion before Tanabe’s new theory, to elucidate why it was considered so radically different. This may be limited to the attitudes of Japanese philosophers, or expand to explicate more generally those ideas in the east and west since the author does discuss that Tanabe’s theory is a combination of eastern and western ideas. It seems there is an ideal space to develop this within the section on the ‘Range of Tanabe’s discussion and religion in Japanese philosophers’.

 Although this may have been beyond the remit of this paper, I would have enjoyed some engagement with some of Tanabe’s earlier theories on the philosophy of religion. The author acknowledges Tanabe as a dynamic practitioner, and in footnote 9 the importance of considering the division of Tanabe’s thoughts into various periods, but some discussion of his earlier attempts to define the philosophy of religion in *Outline of Philosophy* 哲学通論 (1933) could have made an interesting contribution to the paper.

  

- Structure

In this same footnote, footnote 9, the author states that this paper assumes Himi’s position that “Tanabe maintains a consistent problematic awareness of religion, even though the subject matter he addresses changes over time (Himi, op. cit., pp. 19–20)”. Some further discussion of why the author holds this position within the main body of this paper may be beneficial, also because another citation referencing Himi in the main body mentions Tanabe’s philosophy as being “pure” and “thorough” which seems to confuse the author’s position. This critical position is mentioned within the conclusion and very briefly in the “Methods” section rather than having a dedicated place to evaluate this criticism in more depth.

- References

Reference appears to be missing from the third paragraph in the section ‘Absolute religion in Tanabe’ (“is Christianity but is not Christianity”)

 The comment made in the section “Absolute religion in Tanabe”, that the influence of Christian thinkers such as Uchimura Kanzo and Uemura Masahisa etc. was “more significant than those of Buddhist intellectuals such as Kiyozawa Manshi (1863–1903) and Chikazumi Jōkan (1870–1941).” should be more carefully rephrased or better referenced if the author is intending to represent some opinion by Tanabe that this was the case. The statement referenced, that “Christianity “responded even better to the humanist demands of the youth” from THZ 10 pg. 4 does not quite seem sufficient for such a sweeping statement.

- Other comments

Finally, I would like to briefly draw attention to the general desire for philosophers to depart from what could be considered problematic usage of the “east” vs. “west” distinction, which is made a couple of times in the paper. Even though the author might disagree with these kinds of criticisms of characterising philosophy in this way often inspired by Said and McEvilley et al., this should still be acknowledged as a criticism that may crop up, and this distinction isn’t entirely necessary for or central to the fundamental arguments in this paper so can/should be easily avoided. What the author seems to want to say at various points in the paper can be said whilst avoiding this and simply referring to, for example, Buddhist vs. Christian tradition. Making more specific reference to, for example, the distinctions in Buddhist vs. Christian thought not only avoids this criticism of east/west essentialisation but would aid in the clarity, indicating precisely what the author is trying to draw our attention to about this difference. A further argument could be made specifically in the context of Tanabe that this distinction seems counterproductive to his aims and the idea propelled in this paper that Platonism connects “east and west”.

Is the work clearly and accurately presented and does it cite the current literature?

Yes

If applicable, is the statistical analysis and its interpretation appropriate?

Not applicable

Are all the source data underlying the results available to ensure full reproducibility?

No source data required

Is the study design appropriate and is the work technically sound?

Partly

Are the conclusions drawn adequately supported by the results?

Yes

Are sufficient details of methods and analysis provided to allow replication by others?

Yes

Reviewer Expertise:

Japanese philosophy, Tanabe Hajime and Watsuji Tetsuro, philosophy of death and dying, self and identity, world philosophy, community and ethics

I confirm that I have read this submission and believe that I have an appropriate level of expertise to confirm that it is of an acceptable scientific standard, however I have significant reservations, as outlined above.

# Response

**Published:** 2025-02-26

**Authors:** Hiroto Doi

**Affiliations:** University of Tsukuba, Japan

I would like to express my sincere gratitude for the many valuable suggestions given through an accurate peer review. My manuscript contained some narrow-minded descriptions, and your help in improving them was greatly appreciated. I would also like to apologize for the lengthy time interval taken to revise the manuscript after the review.

 The author has been researching Tanabe’s interpretation and criticism of Neoplatonism concerning Plato, which Tanabe cites as his philosophical foundation. This is because the author’s original specialty was researching Neoplatonism from the standpoint of religious studies. In the process, I confirmed that Platonism (from Plato, via Plotinus and others, to Augustine and Eckhart) played a significant role in bridging religion and philosophy in Tanabe. In the manuscript, the author examines how Tanabe’s interpretation of Plato, which bridges philosophy and religion, is involved in the presentation of religion in the present and future, rather than in the past. Tanabe’s understanding of and discussion of Plato should also be examined, but since this is beyond the scope of this study it could be examined again later.

 In the following response, the letters in parentheses is added for organization. The revised version of the manuscript may be made available after this response.

**>** **Recommendations**

**> Content**

**(a) Whether the section on Tanabe as a “practitioner” adds much to the overall article**

 Tanabe’s practice and its role in his theory of religion are certainly significant issues in this manuscript. I added explanations on this point in Notes 16 and 17 and in the third paragraph of the section “4. Tanabe as a ‘practitioner’.” This agrees with the comments of Professor Hoshino, the other reviewer. As mentioned above, Tanabe’s theory of Plato provides clues on this theme.

 Related to (b) below, the uniqueness of Tanabe’s thought is characterized by its emphasis on “practice.” For this reason, the section “4. Tanabe as a ‘practitioner’” was necessary. However, one problem is that it is difficult to understand what kind of practice Tanabe is seeking in the context of religion. He devoted himself to writing philosophic treatises. In general-interest magazines and published works, he addressed the cultured public. Some may not agree that this is Tanabe’s practice about religion, even though his logic would indicate so. However, behind Tanabe’s writing, his treatise as a philosopher was an intense practice that sometimes put him at risk (I have added an episode where he took hostile action against the Japanese government of the time to save K. T. Jaspers). It can be said that this itself is nothing more than a personal ethical practice, and that Tanabe’s understanding and perceptions of the real world are insufficient in some cases. Nevertheless, the author believes that Tanabe’s emphasis on the practice and presentation of his own theory of religion should not be taken lightly.

**> Context and scope**

**(b) Why Tanabe’s theory was considered so radically different**

 An explanation is added at the end of the section “3. The range of Tanabe’s discussion and religion in Japanese philosophers.” The discussion of Christianity, Marxism, and Japanese Buddhism in the *Demonstration of Christianity* is representative of Tanabe’s characteristics in his theory of religion. His philosophy is distinguished by the fact that he mediates between things that negate each other, which reflects his philosophy. This is very different from the theories of religion of other scholars, who tend to focus on the acceptance or criticism of existing religions based on their teachings. From the perspective of theory if religion, it was thought that early Japanese scholars of religion needed to be included in the comparison. Rather than a direct (positive) approach that seeks a common essence, Tanabe’s approach of mediating something utterly different in a negative way is also thought to be suggestive of the religious studies field.

**(c) Tanabe’s earlier theories on the philosophy of religion**

 An addition was made at the end of Note 9. However, there is a certain distance between Tanabe’s philosophy of religion and his theory of religion based on philosophy; therefore, further study may be required.

**> Structure**

**(d) How do we understand consistency and change in Tanabe?**

 This was added in the middle of Note 9. Even though Tanabe adheres to the dialectic of absolute negation, it seems inconsistent that the religion he highly evaluates would change, and it certainly is not easy to understand. As an example of how a change can occur as speculation deepens while maintaining the fundamental principles, I introduced the case of Plotinus and Neoplatonism. In this example, Tanabe was initially critical of Plotinus because he found no negative occasions in him. However, through his reading of W. R. Inge’s work, Tanabe found a negative occasion in Plotinus, leading to a somewhat more favorable interpretation of the latter. In this case, Tanabe’s fundamental principles have not changed either.

**> References**

**(e) Reference appears to be missing from the third paragraph in the section “Absolute religion in Tanabe”**

 As pointed out, there was a fault which I have corrected. Tanabe’s translation in the main text is initially included in the quotation and corresponds to the same THZ 10, p. 319.

**(f) The influence of Buddhist intellectuals and Christian intellectuals**

 The points raised seem to be in common with the response to Professor Hoshino (8). The part indicated was a description based on Tanabe’s recollections; therefore, the sentence was corrected. Additionally, the explanation has been expanded in Note 29. Considering the influence of Chikazumi Jōkan on the Kyoto School philosophers, as Iwata points out, Tanabe’s description seems somewhat exaggerated.

**> Other comments**

**(g) Problematic usage of the “East” vs. “West” distinction**

 The three places where “East and West” were mentioned, including in the abstract, were careless references. Therefore, I changed the text to “Buddhism and Christianity” to suit Tanabe’s context (Abstract and Conclusion), and replaced it with more appropriate explanations (7. Platonism as a framework for world religion in Tanabe). Tanabe does not entirely reject the framework of East versus West, but relying on this framework to discuss his work will likely lead to misinterpretations. This is because, in Tanabe’s works, it is thought that the degree to which dialectics is thoroughly applied is more important than the usual dichotomy of East or West.

 I would like to thank you again for your appropriate and insightful suggestions for improving this manuscript.

# Reviewer report: Reviewer response for version 1

**DOI:** 10.5256/f1000research.143372.r241410

**Published:** 2024-02-26

**Authors:** Seiji Hoshino

**Affiliations:** 1 Kokugakuin University, Tokyo, Japan

**Paper Summary**

 This paper delves into Tanabe Hajime's evolving conception of "religion," particularly during his later years, mainly focusing on Existence, Love, and Practice (1947) and Demonstration of Christianity (1948). While Tanabe has been primarily identified as a philosopher, his philosophical inquiries frequently made references to religious traditions. Scholars have previously documented his shifting interest in historical religions from Shinran (Pure Land Buddhism) through Christianity to Zen Buddhism. The author, however, diverges by focusing on Tanabe's theoretical understanding of "religion" itself, examining Tanabe's use of key terms such as "second religious reformation," "absolute religion," and "world religion." Tanabe posited the notion of a universal religion, which he called "absolute religion," envisioning it as a dialectical synthesis of Christianity, incorporating elements of Pure Land and Zen Buddhism. While acknowledging that this may appear somewhat Christocentric, the author argues that it resulted from Tanabe's rigorous philosophical investigation, particularly his pursuit of the idea of "negation." More specifically, the paper contends that Tanabe's conception of "absolute religion" was deeply rooted in his understanding of Platonism. According to the author, Platonism is a philosophical tradition where the boundaries between philosophy and religion are not distinctly demarcated, and the author concludes that Tanabe's formulation of "absolute religion" emerged from Tanabe's creative interpretation of Platonism.

**Importance of this paper**

 Japanese philosophy of religion has often been described and studied in the context of how it has incorporated Eastern thought into the vocabulary and thinking of Western philosophy. Intensive scholarly works exist on the relationship between Kyoto School philosophers and Buddhist thoughts; Tanabe's understanding of Shinran has been examined. However, this paper argues that Tanabe's conception of "religion" is drawn from his philosophical pursuit and his creative reading of Platonism. The argument makes an interesting case study of Tanabe's thought.

**Suggestions**

 -Context

 That being said, I am a bit wondering how this paper's argument could connect to a broader context. For example, how does Tanabe's conception of religion fit into the history of "religion" discourse in modern Japan? As the author noted that Tanabe's argument would be within "the scope of an intellectual's religion," what role did such an "intellectual's religion" play contemporarily? 

 I understand this is not the main issue the author attempted to address. However, the author could provide his own perspective on the context of Tanabe's "religion." (Perhaps the author could develop his reference to Tanabe's youth or the idea of "shūyō" mentioned in the conclusion.)

 -Tanabe's scope

 I want to make one more point. It is about the readers of Tanabe, or whom Tanabe tried to address; this relates to the question of Tanabe's scope. This paper argues that Tanabe's "absolute religion" involved a certain "practice," namely, a thorough pursuit of philosophical speculation. 

 At first glance, such a "practice" appears to be a personal ethics (for example, Tanabe's residence in Kita-Karuizawa and his attempt at dialogue with Minoda). However, providing that "social practice" and "social improvement" are repeatedly mentioned in the paper, Tanabe's "absolute religion" was, from what I understood, more than a personal ethics. Then, how did Tanabe think his "absolute religion" should be embodied in this world (or, more practically, how should it interact with the actual historical religious traditions of his time)? Of course, Tanabe was a philosopher, not a social scientist; thus, his arguments did not need to be directly connected to the real world. However, the readers might want to know Tanabe's vision regarding this.

 - Regarding the section "Tanabe as a "practitioner"

 Tanabe's "practice" explained in the section is difficult to understand at first read. To grasp the idea in this section, such that Tanabe's "thorough contemplation" was his (religious) "practice," readers need the author's explanation of Tanabe's understanding of Platonism provided before the conclusion. Thus, I suggest the main argument of this section could be moved after the "Platonism as a framework for world religion in Tanabe" section, though this revision requires some rewriting.

 Also, as pointed out above, readers may need an explanation of the relation between Tanabe's "practice" with "social practice" and "transforming society" or of the practical goals of "social practice" in Tanabe's conception.

**Other minor comments**

 - Regarding Notes 12, 13

 The author can provide more explanation regarding the differences between Tanabe's argument of religion and the arguments of the latter half of the 19th century.

 - Regarding Notes 14 and Tanabe's attitude towards "folk religions and practices"

 The author can elaborate on Tanabe's understanding of evolutionary views of religions. Tanabe's attitude towards "folk religions and practices" appears to be influenced by the idea of the evolution of religion. What did Tanabe think about the evolutionary views of religions prevalent in his time? Or was Tanabe's view different from the views of his contemporaries?

 - Regarding the section "Absolute religion in Tanabe"

 "This phenomenon was due to the influence of Christian intellectuals such as Uchimura Kanzō (1861–1930), Uemura Masahisa (1858–1925), and Ebina Danjō (1856–1937). Their influence was more significant than those of Buddhist intellectuals such as Kiyozawa Manshi (1863–1903) and Chikazumi Jōkan (1870–1941)."

 I have some concerns in the author's description above, especially the evaluation of the influence of Buddhist figures because they were also very influential among young intellectuals at that time. If it is Tanabe's retrospect, it is understandable (apparently, those who were influenced by Kiyozawa or Chikazumi would look back differently). Please add citations or revise the description.

 - Tanabe's mention of the second religious reformation

 There is a quote on Tanabe's mention of the second religious reformation (THZ 10, p. 319). The author can elaborate on Tanabe's claim about what points in existing Christianity should be reformed (or negated).

 - Tanabe's views of "religious studies"

 The author cited Tanabe's brief phrase: "relying on today's developments in religious studies" (THZ 10, p. 323). Considering the theme of this paper, the author can explain Tanabe's views of the achievements of religious studies of his time.

Is the work clearly and accurately presented and does it cite the current literature?

Yes

If applicable, is the statistical analysis and its interpretation appropriate?

Not applicable

Are all the source data underlying the results available to ensure full reproducibility?

No source data required

Is the study design appropriate and is the work technically sound?

Partly

Are the conclusions drawn adequately supported by the results?

Yes

Are sufficient details of methods and analysis provided to allow replication by others?

Yes

Reviewer Expertise:

History of religions in modern Japan

I confirm that I have read this submission and believe that I have an appropriate level of expertise to confirm that it is of an acceptable scientific standard, however I have significant reservations, as outlined above.

# Response

**Published:** 2025-02-26

**Authors:** Hiroto Doi

**Affiliations:** University of Tsukuba, Japan

Through careful peer review, I received many valuable suggestions, for which I would like to express my sincere gratitude. I sincerely apologize for the lengthy manuscript revision. This manuscript discusses Japanese philosopher Tanabe Hajime in the field of religious studies, and considerable effort was required to connect different frameworks. Some interesting points were raised during the revision process; however, discussing them in an integrated manner is a future task.

 The numbers in parentheses are for ease of organization. The revised version of the manuscript will be made available after this response.

**> Suggestions**

**> -Context**

**(1) How** **this paper’s argument could connect** **to a broader context**

 This was added to the latter part of the introduction. In the latter half of the 19th century, Japanese religious figures with a background in Christianity or Buddhism presented various discourses on religion. Tanabe’s *Demonstration of Christianity* was published just after World War II, so the timing was somewhat different. However, as stated in the introduction to the book, Tanabe’s theory of religion, while influenced by modern Japanese religious discourse, can be viewed as the future vision of religion to which philosophy has arrived. Therefore, I believe that examining this from the perspective of religious studies is worthwhile. More ambitiously, I hope that the characteristics and significance of modern Japanese religion will be further clarified by combining the study of Japanese philosophy.

**(2) On *shūyō*, mentioned in the conclusion**

 This issue is discussed further in Note 51. The *shūyō* that crosses the boundaries of various religions plays an interesting role in the discourse on religion in modern Japan. I also examined whether a *shūyō* context could be found in Tanabe’s ideas. However, Tanabe rarely used the word *shūyō*, nor did he find the bodily practices that accompanied the *shūyō* of his time, especially before and during World War II. There are several letters written by Tanabe in which he calls for medicines of the time, rather than *yōjō* that does not rely on medicine. Nevertheless, how Japanese philosophers relate to religion and *shūyō* in modern Japan is a meaningful topic, as discussed by Kurita et al. To discuss this, I will need to consider other philosophers in future studies.

**> -Tanabe’s scope**

**(3) Tanabe’s readers, whom Tanabe tried to address**

 I made some additions to the third paragraph of Section 4: Tanabe as a “practitioner.” This problem cannot be avoided when discussing Tanabe’s theory of religion involving practice. Unlike religious figures who called for the reform of their religion or engaged in missionary work, even if the Tanabe’s practice extended into the realm of religion, it is undeniable that Tanabe remained at the level of personal ethics. Even if there is a reason for his own mission as a philosopher, the question remains as to whether this practice has any effect, since he had not gone as far as to practice in society directly but has only stayed at the level of advocacy. This still poses a problem for scholars specializing in philosophy and thought regarding how they should engage in society.

**(4) How** **“absolute religion” should be embodied in this world**

 As Mutō explains, it is thought that Tanabe’s absolute religion and the second religious reformation were intended to be a catalyst for change from within Christianity and Buddhism (THZ 10, pp. 332-334). However, Tanabe did not appear to have specific connections with religious figures. As mentioned in Note 32, he remained in the position of a philosopher, preparing to “wait for the appearance of a religious genius.” However, there are hints in his discussion of religion that Tanabe did not confine himself to a philosophical perspective. The issue of “myth” mentioned at the end of Note 32 may correspond to this; however, I could not fully examine this at this time.

**(5) Tanabe’s practice**

 I added explanations on this point in Notes 16 and 17 as well as in the third paragraph of Section 4. From the perspective of religious discourse in modern Japan, a comparison with Anesaki may be interesting; however, there are some issues regarding their relationship with religious figures and activities in society.

 In addition, as pointed out, Plato is likely the key to approaching this question. It would have been better to discuss this point by reorganizing the sections, as suggested. However, it was difficult to include a sufficient discussion of Plato’s actual practice (rather than interpretations). In addition, because this would have significantly changed the flow of the argument, I avoided changing the structure.

**> Other minor comments**

**> - Regarding Notes 12, 13, and 49**

**(6) Regarding Note 12 and the differences between Tanabe’s argument on religion and the arguments of the latter half of the 19th century**

 The original Note 12 has been revised, and the original Note 13 has been deleted because its content was unclear. Although it is assumed that Tanabe’s involvement in Unitarianism was probably only through books, this is an interesting topic for future research.

**(7) Regarding Notes 14 and Tanabe’s attitude toward “folk religions and practices”**

 This information has been added to Note 13 in the revised manuscript. Tanabe refers to arguments considered as evolutionary views of religion as theories of science and society. However, it seems he did not use them in a theory of religion. However, compared with Nishida, the question remains as to why Tanabe had no interest in religious studies despite his speculations about the future of religion. Although it may be beyond the scope of this study to consider this issue, it is worth investigating.

**> - Regarding the section “Absolute religion in Tanabe”**

**(8) Influence of Buddhist and Christian intellectuals**

 The part indicated was a description based on Tanabe’s recollections; therefore, the sentence has been revised. In addition, the explanation has been expanded in Note 29.

**(9) Tanabe’s mention of the second religious reformation**

 This information has been added to Note 32 in the revised manuscript. This is also partially related to point (4) above.

**(10) Tanabe’s views of “religious studies”**

 This has been added to Note 34 of the revised manuscript. It is assumed that the “religious studies” Tanabe mentions refer to individual studies in theology and Buddhist studies. As mentioned in point (7), it is thought that there is little awareness of “religious studies” corresponding to the current academic field. This suggests that Tanabe’s theory of religion has the problem of not taking folk religions into consideration and also has the characteristic of discussing future religions without being restricted by the framework of pursuing the essence of religion.

 Again, I would like to express my gratitude for the detailed suggestions to improve this manuscript.
